# Peer Review of “Effect of Thermal and Vibration Changes on Automated External Defibrillator Circuit Boards: Finite Element Analysis Study”

**DOI:** 10.2196/80142

**Published:** 2025-08-19

**Authors:** Ayobami Akinfenwa

**Keywords:** circuit board, automated external defibrillator, heart, cardiology, vibration, thermal changes, medical devices

*This is a peer-review report for “Effect of Thermal and Vibration Changes on Automated External Defibrillator Circuit Boards: Finite Element Analysis Study.”*

## Round 1 Review

### General Comments

This paper [1] considered the vibration and thermal analysis of a modeled circuit board of an automated external defibrillator (AED) using Ansys. The vibration failure in the modeled circuit board with four rigid supports was pronounced starting at the taller components, including the capacitor. The board was reinforced to an 8–rigid support system, reducing the failure around the rigid supports. The thermal failure started from the battery position, causing thermal dissipation to other parts of the board, ultimately leading to the failure of the circuit board and the AED.

### Specific Comments

#### Major Comments

1. “The goal is to analyse the effect of vibration and thermal experience on the AED based on its operation.” Is this your research statement? I suppose the topic suggests you analyzed the modeled circuit board, not the overall AED system. If not, how did you measure the overall effect on the AED?

2. The author may also need to discuss the importance of the circuit boards in an AED in the Introduction.

3. The figures will need a little more discussion.

#### Minor Comments

4. “Vibration and Thermal Analysis on Modeled Circuit Board of Automated External Defibrillator (AED) Medical Device” will likely communicate the title better.

5. I find it a bit difficult to understand this line: “Fatigue failure under sinusoidal vibration loading for component by comparing the vibration failure test, FEA, and theoretical test (Y.S.Chen, 2008).” What did you want to say?

6. Figure 1 will need relabeling. The labeling seems to cover some parts of the board. A transparent background could help.

## Round 2 Review

1. The recommendation about explaining how the author measured the overall effect of the analysis on the AED or the board and the recommendation that the author should provide more evidence on how the 4-member and 8-member supports affect the analysis result have not been answered or addressed in the manuscript. The author should consider these.

2. The author will also need to be consistent. Is Figure 5 the same as Figure 5 or Fig. 5? It should be corrected for all other instances.

3. Additional citations might be needed in the work; it still looks like over 40% of the citations are 15 years or older. Also, “et al.,” should be in italics with a period after the “al.” The Discussion also seems not well discussed in relations to previous works.

4. The template format should also be considered carefully.

## Abbreviations

AED: automated external defibrillator
